# Prognostic Value of Discs Large Homolog 7 Transcript Levels in Prostate Cancer

**DOI:** 10.1371/journal.pone.0082833

**Published:** 2013-12-09

**Authors:** Christian R. Gomez, Farhad Kosari, Jan-Marie Munz, Claire A. Schreiber, Gaylord J. Knutson, Cristiane M. Ida, Abdelouahid El Khattouti, R. Jeffrey Karnes, John C. Cheville, George Vasmatzis, Stanimir Vuk-Pavlović

**Affiliations:** 1 Stem Cell Laboratory, Mayo Clinic Cancer Center, Mayo Clinic, Rochester, Minnesota, United States of America; 2 Division of Hematology, Department of Internal Medicine, Mayo Clinic, Rochester, Minnesota, United States of America; 3 Division of Preventive and Occupational Medicine, Department of Internal Medicine, Mayo Clinic, Rochester, Minnesota, United States of America; 4 Department of Molecular Medicine, Mayo Clinic, Rochester, Minnesota, United States of America; 5 Department of Laboratory Medicine and Pathology, Mayo Clinic, Rochester, Minnesota, United States of America; 6 University of Mississippi Medical Center, Jackson, Mississippi, United States of America; 7 Department of Urology, Mayo Clinic, Rochester, Minnesota, United States of America; National University of Ireland Galway, Ireland

## Abstract

Hypoxia has been associated with malignant progression, metastasis and resistance to therapy. Hence, we studied expression of hypoxia–regulated genes in 100 prostate cancer (CaP) bulk tissues and 71 adjacent benign tissues. We found 24 transcripts significantly overexpressed (p≤0.02). Importantly, higher transcript levels of disc large (drosophila) homolog-associated protein 5 (DLGAP5)/discs large homolog 7 (DLG7)/hepatoma up-regulated protein (HURP), hyaluronan-mediated motility receptor (HMMR) and cyclin B1 (CCNB1) were associated with higher Gleason score and more advanced systemic progression. Since the products of HMMR and CCNB1 have been identified recently as molecular markers of CaP progression, we postulated that DLG7 has prognostic value too. To test this hypothesis, we measured transcript levels for DLG7 in a 150-pair case-control cohort. The cases (progression to systemic disease within six years of surgery) and controls (no progression within eight years) were matched for clinical and pathologic prognostic variables, including grade, stage, and preoperative serum levels of PSA. The overall prognostic ability of DLG7, as tested in receiver operating characteristic analysis was of 0.74 (95% CI, 0.68 to 0.8). Overall, our data indicate that expression of DLG7, a hypoxia-controlled gene, holds prognostic potential in high-risk CaP; this also demonstrates that variation of oxygen tension may constitute a tool for identification of novel biomarkers for CaP.

## Introduction

The role of oxygen tension (*potentia oxygenii*, *p*O_2_) in tumor biology has been unappreciated for a long time; a reason for it has been the relative oxygen-independence of tumors (“the Warburg effect”; ref [1].) that led to the assumption of tumor insensitivity to changes in oxygen tension. More recently, tumor–associated hypoxia has been associated with malignant progression, metastasis, resistance to therapy, and poor clinical outcome [2,3]. In common with other solid tumors, *p*O_2_ in prostate cancer (CaP) fluctuates, giving rise to acute and chronic hypoxia [4]. Some claim that low *p*O_2_ is an independent indicator of poor clinical outcome for CaP patients [5], but *p*O_2_ values measured in the CaP nidus do not always correlate with clinical outcome [4] suggesting the need for more relevant hypoxia-associated biomarkers of aggressive CaP.

Currently used markers that predict outcome in men with CaP include the Gleason score, TNM stage, surgical margin status, and preoperative serum levels of prostate-specific antigen (PSA) [6-8]. Whereas stratification by these variables often effectively predicts the course of disease, tumors with similar biochemical, histopathologic, and clinical conditions can still behave very differently. Recently, transcriptome studies of CaP identified several genes associated with disease outcome. Among them, expression of hypoxia-regulated molecules [i.e., vascular endothelial growth factor (VEGF), hypoxia-inducible factor (HIF)-1, osteopontin, lysyl oxidase (LOX) and glucose transporter-1 (GLUT-1)] correlated with pathological status and patient features (reviewed in ref [4].). These studies show the feasibility of identifying biomarkers linking CaP, hypoxia and prognosis and establishing the contribution of hypoxia-associated genes to CaP progression. Identification of hypoxia-related biomarkers might help identify the patients who could benefit from hypoxia-targeted therapies [4]. Here we report the study of transcription of genes, previously found under hypoxia control, in CaP patients. Our data demonstrate that hypoxia-sensitive genes have a prognostic value in high-risk CaP and that variation of oxygen tension may constitute a tool for further identification of biomarkers for CaP.

## Materials and Methods

### Prostate cancer transcriptome

All studies in this report were approved by the Mayo Clinic Institutional Review Board. In general, we followed our previously described method [9]. Briefly, we used frozen tissue to study the transcriptome in cancer cells isolated by laser-capture microdissection (LCM) or without isolation (“bulk tissue”). Samples were obtained from non-neoplastic prostate tissue, primary prostate cancer, and prostate cancer metastases. Details of frozen specimen collection, preservation, sectioning, hematoxylin and eosin staining, pathological assessment, LCM, analysis of RNA integrity, and linear amplification have been published earlier [10]. We obtained and analyzed the transcriptome of 102 LCM-isolated prostate cancer cell samples; 19 samples of non-neoplastic prostate epithelium adjacent to tumor; 10 samples of benign prostatic hyperplasia (BPH); and five samples of high-grade prostatic intraepithelial neoplasia (HGPIN). Tumor samples included Gleason pattern (GP)3 cells from 28 Gleason score (GS)(3+3) patients and three GS(3+4) patients; GP4 cells from 10 GS(4+4) patients, 4 GS(4+3) patients, and 6 GS(4+5) patients; and GP5 cells from 5 GS(5+5) patients and 5 GS(5+4) patients. Tumor samples included also seven CaP metastases from lymph nodes. Expression profiles of bulk samples (n = 37) were obtained from high-risk patients with GS7 and higher; these tumors were independent from those subjected to cell isolation by LCM. The samples included tumors from 18 patients whose disease progressed systemically and who died in less than six years after surgery (aggressive disease) and 19 patients who survived more than eight years following surgery (non-aggressive disease). Disease outcome was not revealed to researchers on this study before the completion of experiments and conclusion of all analysis. 

Probe set expression values were obtained from the raw microarray data (.cel files) using the gcrma package in the R Project for Statistical Computing (http://www.r-project.org/). For further analysis we selected the probes overexpressed twofold or more in CaP above the mean expression levels in non-neoplastic prostate tissues and BPH tissues, both in bulk and in LCM–isolated cells.

### Case-control matching of men with high-risk prostate cancer

One hundred fifty men whose CaP systemically progressed (SP, documented by biopsy or radiographic proof of metastases) or who died with metastatic CaP within six years of prostatectomy were identified in the Mayo Clinic Radical Prostatectomy database for years 1994 to 2004. Tissue samples for those patients were archived; evidences on biomarker discovery and validation projects for this study group have been previously published by us [9,11,12]. A computerized system [7] matched these patients with 150 control men whose disease did not progress and who did not die for at least eight years of follow-up; matching criteria included Gleason score, TNM stage, margin status, preoperative PSA, and GPSM score (Table 1). Multivariate analysis that included DLG7 and any of the clinical parameters did not add to the accuracy of the model compared to a model that included DLG7 alone. Specimens were blinded for clinical status and reviewed by J.C.C., a board-certified pathologist. Of the total of 300 samples, 141 SP samples and 117 control samples were selected by pathology criteria and by the fact that they provided sufficient tissue of the highest Gleason pattern for experimental analysis. For SP patients the median follow-up from radical prostatectomy to progression or last follow-up was 2.4 years; for control patients the median follow-up was 13.2 years. 

**Table 1:** Clinical parameter values in cases and controls.

	**controls**	**Cases**	**p-value †**
**Pathological Gleason score (GS)**			0.77
GS5	4	2	
GS6	5	4	
GS7	61	82	
GS8	23	17	
GS9	37	38	
GS10	1	2	
**TNM stage**			0.46
T2aN0	12	9	
T2bN0	27	21	
T3aN0	20	33	
T3b4N0	34	54	
TxN+	29	28	
**Margin status**			0.54
0	45	55	
1	86	90	
**Preoperative serum PSA**			0.67
mean (range) ng/ml	19.0 (0.9 - 119)	20.1 (1.3 - 143)	
**GPSM**			0.56
mean (range)	11.5 (6.0 - 16.0)	11.7 (6.0 - 16.0)	

^†^ p-values refer to the comparison of cases and controls.

### RNA extraction and real-time PCR for the case-control study

Hematoxylin and eosin-stained tumor sections obtained from formalin-fixed paraffin-embedded blocks were blinded with regard to whether they belonged to SP samples or control samples; the sections were reviewed by pathologists C.M.I. and J.C.C. who assigned the primary and secondary, but not tertiary Gleason patterns. The block assigned the highest score for the primary or secondary Gleason pattern and containing the most tumor tissue of that pattern was selected for further analysis. Each block was sectioned into 10-μm thick sections. Tumor tissue was deparaffinized in xylene and scraped from the slide under RNase-free conditions into a 1.5-mL tube containing digestion buffer (RecoverAll kit, Ambion, Carlsbad, CA). Details about RNA extraction from paraffin blocks and quality control have been published earlier [9]. Total RNA was isolated according to the RecoverAll procedure and treated with DNase using Ambion Turbo DNA-free Kit according to the manufacturer's instructions. RNA was quantified with the Quant-iT RiboGreen kit (Invitrogen, Carlsbad, CA). Quantitative PCR was used as reported earlier [11] to assess transcript levels for the discs large homolog 7 (DLG7) [a.k.a. disc large (drosophila) homolog-associated protein 5 or hepatoma up-regulated protein], hyaluronan-mediated motility receptor (HMMR) and cyclin B1 (CCNB1). We reversely transcribed 500 ng RNA in 40-μL reaction volume by the use of Superscript III First Strand Synthesis System (Invitrogen). Primers for quantitative PCR were designed by the use of Primer Express software (Applied Biosciences, Carlsbad, CA) to amplify a 70-bp to 85-bp fragment identical to the target sequence on the Affymetrix microarray. Differences in cycle threshold, Δ(*Ct*), were obtained by subtracting the *Ct* value of the normalizing gene from the *Ct* value of the test gene of the same reverse transcription reaction. The normalizing gene, 40S ribosomal protein S28 (RPS28), was chosen previously as the most stably expressed in all normal and CaP samples [12]. Levels of DLG7 and RSP28 transcripts in 22 aggressive and 32 non-aggressive tumors were not determined because of insufficient RNA quality. 

### Statistical methods

We analyzed the data with the R package (http://www.r-project.org/). Distributions of clinical and pathologic parameters were compared by the χ^2^ and *t* tests. Receiver operating characteristic (ROC) curve areas were estimated for clinical and pathologic parameters for all patients. Standard errors of the mean for areas under the curves were computed by the rank correlation for censored data (rcorr.cens) test. 

## Results

### Hypoxia-controlled transcripts are associated with disease stage and prognosis

**Figure 1:**
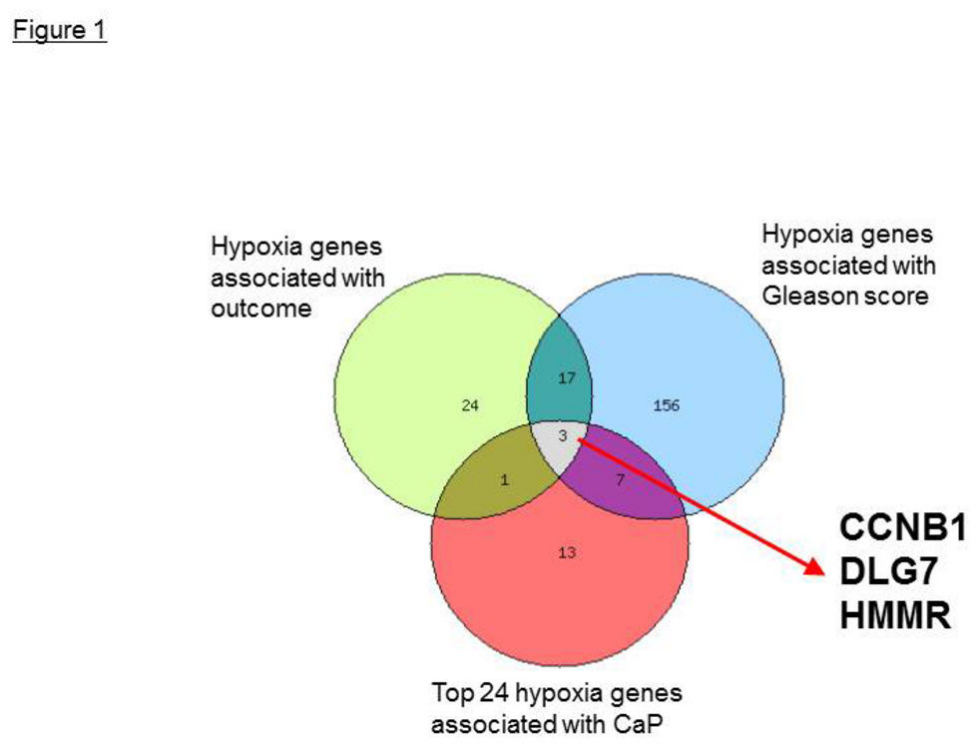
Three hypoxia-controlled genes associated with Gleason score and prognosis. Among the hypoxia-regulated genes significantly overexpressed in CaP, cyclin B1 (CCNB1), DLGAP5 and hyaluronan-mediated motility receptor (HMMR) were associated with Gleason score and disease outcome.

Because CaP appears characterized by a hypoxic transcriptome [13-15], we hypothesized that hypoxia–regulated genes will provide additional insight into the mechanisms of CaP progression. We tested this hypothesis by complementary data mining [16]. We analyzed the 88 hitherto known transcripts regulated by hypoxia-inducible factor 1 (HIF-1) [17]; 500 hypoxia-associated genes identified in cell lines [17]; 23 genes of the conserved core hypoxia signature [18]; twelve HIF-1 targets tested in CaP [13]; and 708 genes in the Ingenuity hypoxia signaling pathway [19]. Among all hypoxia-regulated genes, in our samples we identified 24 genes significantly overexpressed in CaP by at least twofold *both* in bulk tissue and LCM–isolated cells (*p*≤0.02; Table 2). Importantly, transcript levels for DLG7, CCNB1, and HMMR were higher in samples characterized by the higher Gleason score and in patients with poorer prognosis (Figure 1). We described the prognosis for the patients in this study earlier [9].

**Table 2:** Hypoxia-associated genes significantly overexpressed in CaP bulk tissue and samples isolated by LCM.

		**Bulk tissue**		**LCM**	
**Symbol**	**Name**	**CaP/N log_2_ ratio**	**p value**	**CaP/N log_2_ ratio**	**p value**
ACACA	acetyl-Coenzyme A carboxylase alpha	1.5	0	1.2	0.002123
CDCA3	cell division cycle-associated protein 3	1.7	0	1.5	0
CEP55	centrosomal protein 55kDa	1.8	0	1.4	0
CCNB1	cyclin B1	1.8	0	1.3	0
CKS2	cyclin-dependent kinases regulatory subunit 2	1.1	0.000043	1.2	0.000001
DLGAP5	discs large homolog 7 (DLG7)	1.8	0	1.5	0
SLC7A1	high affinity cationic amino acid transporter 1	1.6	0	1.2	0.000023
HMMR	hyaluronan-mediated motility receptor	2.1	0	1.7	0
HIG2	hypoxia-inducible protein 2	1.5	0	1.2	0.000001
LOX	lysyl oxidase	1.1	0.000044	1.5	0
MMP10	matrix metalloproteinase-10	1.2	0.021521	1.4	0.000066
MCOLN2	mucolipin 2 (cation channel protein)	1.1	0.000332	1.4	0
NLN	neurolysin (metallopeptidase M3 family)	1.2	0	1.2	0.000012
PDLIM5	PDZ and LIM domain 5 (Scaffold protein)	1.1	0	1.2	0.000121
PSD3	pleckstrin and Sec7 domain containing 3	1.1	0.000001	1.2	0
C20orf74	Ral GTPase-activating protein subunit alpha-2	1.3	0	1.2	0.000092
FAM80A	ribosomal modification protein rimK-like family member A	1.7	0	1.2	0.000013
SDK1	sidekick homolog 1, cell adhesion molecule	1.5	0	1.5	0
STC2	stanniocalcin-2 (secreted)	1.2	0.009053	1.3	0.000059
SOX4	transcription factor SOX-4	1.2	0	1.2	0.000001
TMEM200A	transmembrane protein 200A	1.3	0	1.3	0.000886
TFF3	trefoil factor 3 (intestinal, stable secretory protein)	1.2	0.001889	1.2	0.001276
UBE2C	ubiquitin-conjugating enzyme E2 C	1.4	0	1.2	0
UBE2E3	ubiquitin-conjugating enzyme E2 E3	1.8	0	1.3	0.000079

Abbreviations: CaP, prostate cancer; N, normal; LCM, laser capture microdissection

**Figure 2:**
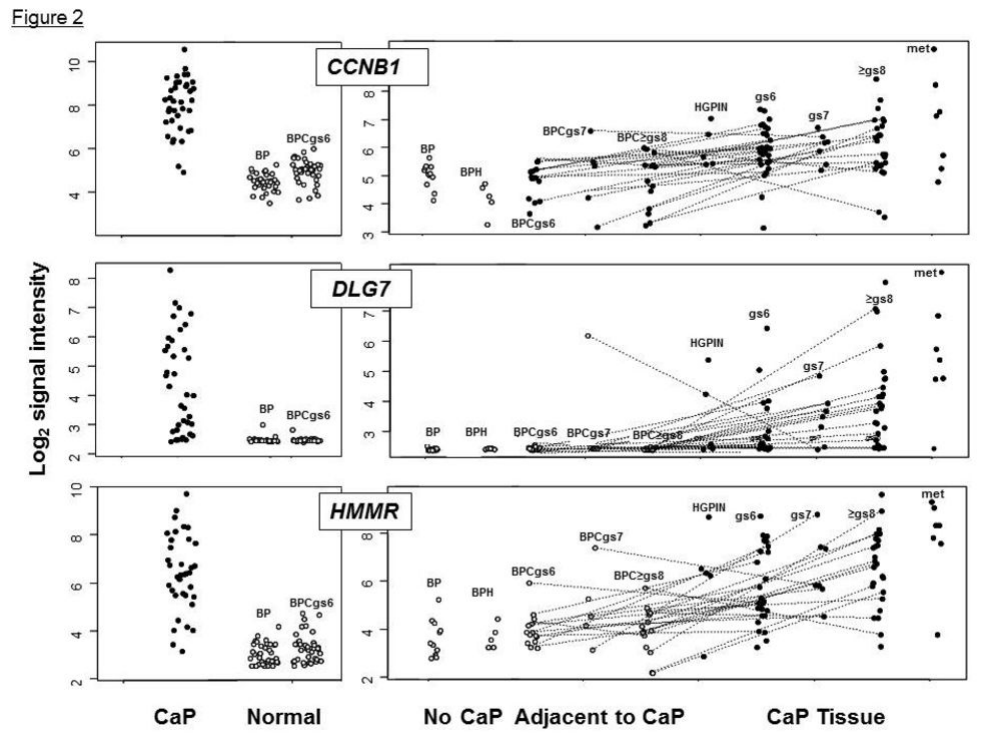
Transcript levels for CCNB1, DLG7, and HMMR measured in CaP and noncancerous prostate tissue. Panels on the left compare transcript levels in CaP bulk tissue (full symbols) with the levels measured in benign prostate tissue (open symbols) from men free of CaP (BP) and in benign prostate tissue (BPC) adjacent to CaP of combined Gleason score 6 (gs6). Panels on the right display transcript levels measured in non-neoplastic prostate epithelial cells isolated by laser capture microdissection (LCM) in benign tissues (open symbols): BP, benign prostatic hyperplasia (BPH) and BPC adjacent to CaP of the indicated Gleason score (gs). Full symbols in panels on the right denote transcript levels measured in LCM-isolated CaP cells: high-grade prostatic intraepithelial neoplasia (HGPIN), the cells isolated from areas of combined Gleason scores 6 through 8 and cells isolated from lymph node metastases (met). CCNB1, cyclin B1; DLG7, discs large homolog 7; HMMR, hyaluronan-mediated motility receptor.

Figure 2 shows the transcript levels for CCNB1, DLGAP5, and HMMR genes at different disease stages. Both in tumor tissue and LCM–isolated cells, transcript levels were higher in CaP than in normal tissue. Also, these transcripts were expressed at significantly higher levels in the cells isolated from CaP tissues at stages GP4 and GP5 than at GP3 stage, suggesting an association with tumor progression. Furthermore, transcripts levels were higher in metastases from patients who progressed to death, suggesting an association with disease outcome (see Methods) (Figure 2).

The products of CCNB1 [20] and HMMR genes [16] have been recently identified as molecular markers of CaP progression. Therefore, we posited the potential utility of hypoxia–associated genes as CaP biomarkers with prognostic value. To scrutinize this idea, we correlated the DLG7 transcript levels with those of DNA topoisomerase 2α (TOP2A), a gene whose transcripts are of prognostic importance in CaP [21], particularly in the high-risk form [11]. We found a high correlation between the levels of the two transcripts (Pearson coefficient=0.816). The revealing of this association prompted us to hypothesize that DLG7 transcripts could independently predict disease outcome in men with high risk CaP.

### DLG7 transcripts as potentially an independent predictor of CaP outcome

**Figure 3:**
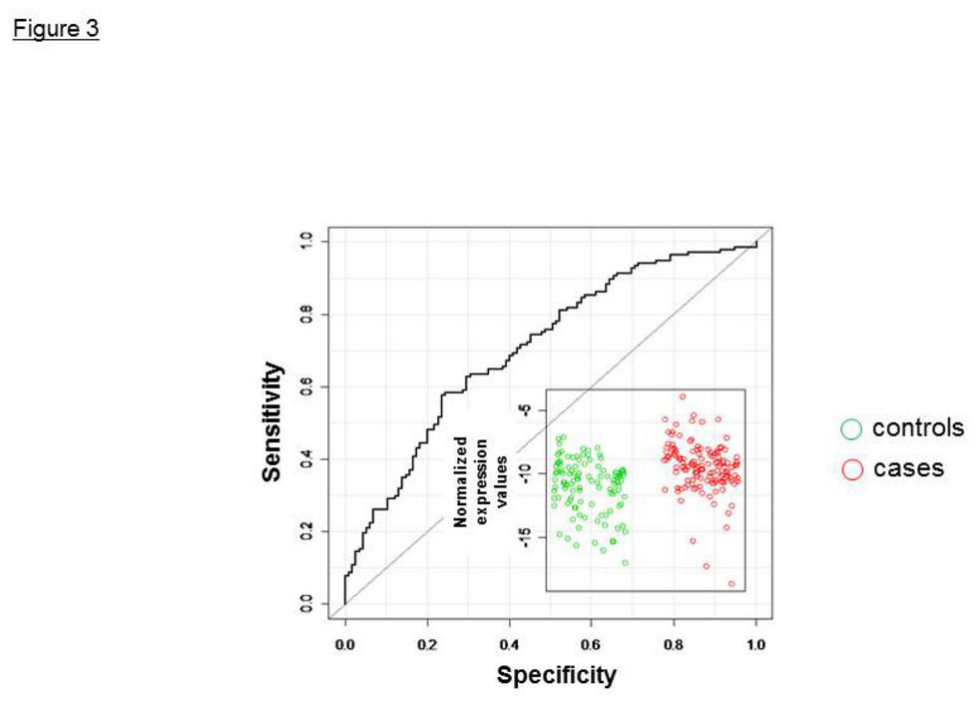
Receiver operating characteristic (ROC) analysis for DLG7. The area under the curve was 0.74 (95% CI, 068–0.80). Inset: Scatter plot of the normalized expression values for cases (red) and controls (green).

We examined the association of DLG7 transcript with systemic progression and death by CaP in a 150 pair case-control cohort [11]. The cases and controls were closely similar in clinical and pathologic features with the exception of DNA ploidy [11]. By ROC analysis we found the area under the curve (AUC) for DLG7 of 0.74 (95% CI, 0.68 to 0.8); for HMMR of 0.67 (95% CI, 0.60 to 0.74); and for CCNB1of 0.64 (95% CI, 0.57 to 0.71). ROC scores for DLG7 demonstrate the overall separation of cases and controls (Figure 3). Thus, we conclude that DLG7 transcript levels predict disease outcome in men at high risk from CaP. In addition, we found that transcript levels of hypoxia-controlled genes CCNB1 and HMMR—previously identified as molecular markers of CaP progression—were associated with Gleason score and disease prognosis as well. 

## Discussion

Gene expression patterns have been used as a tool to identify prognostic markers for CaP [22-26]. Earlier, we and others have proven the prognostic value of selected transcripts for men undergoing radical prostatectomy [9,11,12,27]. In this communication we show that the transcripts of DLGAP5, a hypoxia-associated gene [17], have a prognostic value in high-risk CaP.

Although quantifying *p*O_2_ in CaP tissue *in situ* is at a preliminary stage [4,28], hypoxia has been an independent indicator of poor outcome [5] associated with clinical and pathological variables [28]. Importantly, transcript levels of hypoxia-responsive molecules [i.e., VEGF, HIF-1α, osteopontin, LOX and GLUT-1] are positively correlated with pathology and aggressiveness (reviewed by Stewart et al., ref [4].). These studies demonstrate the feasibility of identifying biomarkers linking hypoxia and prognosis in CaP and establishing the contribution of hypoxia-associated genes to CaP progression.

Because of the apparent association of the hypoxic transcriptome and CaP [13-15], we assumed that hypoxia–associated genes could provide additional insights into the mechanisms of CaP progression. By data mining we identified hypoxia–associated genes with expression significantly modified in CaP, both in bulk tissue and CaP tissue isolated by LCM. Of these genes, we found that transcript levels of DLGAP5, CCNB1, and HMMR were associated with Gleason score and systemic progression. Remarkably, the association between transcription and disease outcome was not observed for other hypoxia-controlled genes previously reported as potential prognostic biomarkers (Lox, ref [13].; Table 1) thus suggesting the potential of transcripts for DLG7, CCNB1 and HMMR as hypoxia–regulated biomarkers specific for CaP.

Since the products of the CCNB1 and HMMR genes have been previously associated with transformed cells and proposed as markers of poor prognosis for numerous malignancies [29-33] including CaP [16,20], we focused on DLGAP5. The DLGAP5 gene encodes a cell-cycle-regulated, microtubule-associated protein known as DLG7, DAP-5 or HURP [34] that acts as a Ran GTPase effector involved in stabilization of the mitotic kinetochore fiber [35]. In hepatocellular carcinoma [36], meningioma [37] and adrenocortical tumors [38] the levels of transcripts encoding DLG7 increased with disease aggressiveness. These transcripts were detected in liver and colon tumors, but not in normal adjacent tissues suggesting an association of DLG7 and carcinogenesis [39,40]. However, information on DLG7 in urological diseases and particularly in CaP is limited [34]. In one study, transcripts for DLG7 were detected in nearly 90 percent of transitional cell carcinoma (TCC) of the bladder, but not in benign urological diseases; a high level of transcripts for DLG7 was found in recurrent TCC [41]. 

In the light of information suggesting DLG7 as a prognostic marker, we explored its potential as outcome predictor in high-risk CaP. Hence, we analyzed the correlation of transcript levels for DLG7 and TOP2A because the prognostic value of TOP2A in CaP has been well documented [21,26,42]. In particular, we reported that TOP2A transcripts [11] and the protein product [12] were prognostic of high-risk CaP. We found that transcript levels for DLG7 and TOP2A were highly correlated and suggest that DLGAP5 gene transcription should be further studied for its potential as predictor of outcome in high-risk CaP. Of note, overexpression of transcripts for TOP2A and DLG7 has been recently identified in grade III meningioma in comparison to grade I meningioma [37]. This information adds value to the notion that co-expression of TOP2A and DLG7 is relevant in tumorigenesis and should be further explored in the search of biomarkers for advanced disease.

To provide the possibility of testing the prognostic value of DLG7 in high-risk CaP within the context of other prognostic transcripts, we investigated the patients we studied earlier [9,11,12]. Men who did and did not systemically progress or die of CaP were matched on Gleason score, TNM stage, margin status, and preoperative serum PSA. The AUC for DLG7 was 0.74 (95% CI, 0.68 to 0.8). Interestingly, the AUC for DLG7 was similar to the value reported for TOP2A alone (0.71; ref [11].). ROC for HMMR [0.67 (95% CI, 0.60 to 0.74)] and CCNB1 [0.64 (95% CI, 0.57 to 0.71)] revealed lower AUC values for these genes than for DLG7. For this reason we did not include HMMR and CCNB1 in a prognostic cancer model. Nevertheless, when compared to HMMR and CCNB1, DLG7 exhibited a higher AUC suggesting a superior prognostic value for high-risk CaP.

Biology of the DLGAP5 gene is compatible with the involvement in cancer formation and progression and suggests that the gene and its product may be potential therapeutic targets. DLGAP5 transcription rises during the S-phase and is maintained at both G2-phase and M-phase of the cell cycle [43]. DLG7 localizes at spindle poles during mitosis [34], hyperstabilizes the mitotic spindle and activates the spindle checkpoint, thus exerting control on spindle stability throughout the cell cycle [44]. Recent data in ovarian cancer cells indicate that DLG7 is a direct downstream target of Notch 3 [45]. This information adds the Notch pathway to the molecular signaling networks controlling DLG7 expression and provides a novel avenue worth pursuing when exploring the possibilities for therapeutic targeting of DLG7. When overexpressed in 293T cells, DLG7 enhanced cell growth at low serum levels and enhanced colony formation [34]. Similar results were found in NIH3T3 embryonic fibroblasts overexpressing DLG7 [46]. More recently, it has been proposed that DLG7 is oncogenic by its effect of reducing the levels of tumor suppressor p53 protein [47]. Overexpression of DLG7 in NIH3T3 cells enhanced the susceptibility to deoxycytosine analogs [46]; therefore, exploring the targeting of DLG7 (for example, by intervening with the pathways controlling DLG7, like those influenced by aurora-A kinase or Notch) could enhance the efficacy of chemotherapeutics in castration–resistant CaP. These observations support the role of DLG7 in cancer progression and propose it as a potential therapeutic target worthy of further research.

Although the role of hypoxia in DLG7 expression has not been demonstrated directly, it has been inferred by the finding that DLG7 is a Notch 3 target in ovarian cancer cells [45]. It has been shown that hypoxia controls transcription of Notch targets in neuroendocrine differentiation of human CaP cells [48]; this implies the possibility of Notch signaling in hypoxia-mediated control of DLG7 expression in CaP and its role in the manifestation of tumor aggressiveness. To look for the potential mechanistic basis of DLG7 sensitivity to hypoxia, we searched the promoter region of DLG7 for the binding sites of the transcription factor HIF. Using the published information on the minimal *cis*-regulatory elements required for HIF–dependent transactivation [49,50] we found five putative HIF binding sites located 1134, 2498, 2616, 3815, and 8310 nt upstream of the DLG7 transcription start, respectively (CRG and AEK, unpublished data). Most of these sites are located in a highly methylated region; since promoter methylation regulates expression of hypoxia-controlled genes in a context-dependent manner [50], the effects of oxygen tension on the expression of DLG7 deserve more investigation.

Our results validate the transcripts of DLGAP5, a *p*O_2_-regulated gene [17], as an independent prognostic biomarker in CaP. Its potential applications include the refinement of the current prognostic tools for CaP and better predictors of therapeutic outcome. The evidence of substantial sensitivity of CaP cells to hypoxia might lead to enhanced efficacy of therapy not only for CaP, but it can also serve as a paradigm for other forms of cancer.
